# Involuntary Vocalisations and a Complex Hyperkinetic Movement Disorder Following Left Side Thalamic Haemorrhage

**DOI:** 10.1155/2003/980839

**Published:** 2003-01-01

**Authors:** T. Dietl, D. P. Auer, S. Modell, C. Lechner, C. Trenkwalder

**Affiliations:** Max Planck Institute of PsychiatryKraepelinstr. 1080804 MunichGermany

**Keywords:** vocalisation, thalamus, palilalia, hyperkinesia

## Abstract

A variety of involuntary speech phenomena as for example palilalia have been described as consequences of neurological disorders. Palilalia is the involuntary repetition of syllabels, words and phrases in ongoing speech. We describe a 73 year old woman who suffered from a hypertensive thalamic haemorrhage. MRI revealed that the lesion was predominantly located within the pulvinar, extending to the lateroposterior thalamic nuclei and to the pretectal area with possible involvement of the medial geniculate body. Few months after the event she developed involuntary vocalisations with whole words and meaningless syllables being rapidly reiterated. In contrast to typical palilalia these vocalisations were not meaningfully related to the ongoing speech of the patient. In addition, the patient developed a complex hyperkinetic movement disorder with right-sided painful hemidystonia and bilateral clonic jerks and a right-sided postural tremor.

## 1. Introduction

Among the most striking consequences of brain diseases is the release of complex involuntary speech phenomena beyond the intentional control of the affected subject. A rich phenomenology of such disorders has been described [5,7,22].

Palilalia is the involuntary repetition of syllables, words or phrases of the ongoing discourse. It was recognized as a comparably rare speech disorder associated with various basal ganglia diseases (e.g. postencephalitic parkinsonism, progressive supranuclear palsy or Gilles de la Tourette syndrome) or disorders of the frontal lobes [5,11,19]. The repetitions are often uttered with increasing rapidity and decreasing voice volume (*palilalia aphone*). Related to palilalia is the phenomenon of logoclonus as the compulsive repetition of the last syllable of a word. Echolalia is the involuntary repetition of utterances of other persons. Vocal tics in the Gilles de la Tourette syndrome and related tic disorders range from simple noises as throat-clearing or sniffing to highly organized verbal utterances as words or short sentences. In Gilles de la Tourette syndrome palilalia and echolalia are common and are then regarded as vocal tics [12]. Klazomania is a scream-like vocalisation described in postencephalitic states [2,3].

Though the thalamus has been appreciated as a crucial subcortical structure in the mediation of speech and language [17] few reports focus on the role of the thalamus in involuntary speech phenomena: Abe et al. [1] described a patient with a ‘repetitive speech disorder’ as a consequence of infarction of the paramedian thalami and midbrain. This patient showed involuntary repetition of syllables, mainly the first syllable of a word, in spontaneous speech. The authors likened this speech disorder to stuttering (‘stuttering like repetition’).

Yasuda et al. [23] reported on a case of palilalia following an infarction of the medial thalami, subthalami and midbrain. This patient showed involuntary palilalic repetitions, mainly of syllables and words, up

ISSN 0953-4180/03/$8.00 © 2003 – IOS Press. All rights reserved to five to ten times in spontaneous speech. The speech rate was reported to increase during repetition with a decrease in voice volume resulting in whispering vocalisations (palilalia aphone). Fung et coworkers [9] described a patient with involuntary rapid reiterations of sentences and complex movements following basilar artery thrombosis with infarction involving the dorsomedial and posterolateral nuclei of the right thalamus and the occipital lobe.

We report on a patient with involuntary vocalisations and a complex hyperkinetic movement disorder developing after left thalamic haemorrhage.

## 2. Case report

A 73 year old woman without any prior history or known family history of a movement disorder suffered from a hypertensive thalamic bleeding with ventricular haemorrhage one year before admission to our hospital. A transient aphasic disorder with mild word-finding difficulties and right sided hemiparesis were the immediate consequences of the lesion. The patient was able to walk short distances without any aid. There was no evidence for dysphasic or dysarthric language or speech impairment.

About three month later she started experiencing bouts of uncontrollable staccato-like vocalisations. On admission there were rapid reiterations of whole words (like 'Katzekatzekatze. . . (cat)) during the neurological examination without a meaningful relation to the present situation). In the further course compulsive rapid repetition of meaningless syllables (like 'talawtalawa. . . ') were predominant. Introspective account of the vocalisations by the patients was poor and she tried to dissimulate this phenomenon. The speech was well articulated without any evidence of dysarthrophonia. Though the involuntary repetitions were rapid there was no increase in speech rate with diminishing voice volume (*palilalia aphone*).

The patient was fully oriented and of clear consciousness during this rapid reiteration of syllables. The vocalisations occurred spontaneously and interfered with ongoing speech. The patient was considerably distressed by her struggle to utter intentional communicative speech in the face of interference of unintentional vocalisations.

At about the same time as the vocalisations started she had developed right-sided painful hemidystonia with involuntary dystonic movements of the neck and the right arm. In addition she had sudden clonic jerks predominantly in the contralesional right arm, but also in the left arm and developed a right-sided postural tremor. Abnormal movements and involuntary vocalisations were markedly exacerbated by stress.

The neurological examination revealed intact cranial nerves including full range of eye movements. Muscular tone was increased at the right body half with normal muscular power. Reflexes were brisk and symmetrical. The plantar response was flexor. There was evidence for a mild right-sided hemihypesthesia.

The MRI showed the chronic stage of a left-sided thalamic haematoma with hemosiderin deposition, cyst, gliosis and atrophy. The lesion was predominantly located within the pulvinar but extending anterior-laterally to the lateroposterior thalamic nuclei and medio-caudally to the pretectal area with possible involvement of the medial geniculate body. There was unilateral hyperintensity of the corona radiata most likely representing secondary Wallerian degeneration.

EEG showed mild background slowing and increased beta-activity probably due to the treatment with benzodiazepines.

Repeated EEGs showed no evidence for epileptic discharges nor was there any clinical evidence for seizures.

## 3. Discussion

### *3.1. Vocalisations*

The most unusual feature of the disorder of this patient are the involuntary vocalisations. The rapid repetition of syllables or words would be consistent with palilalia. However, involuntary repetitions emerged without apparent relationship to ongoing speech and were seemingly out of context. Thus, the vocalisations appeared to have a 'productive' quality and can therefore not be accounted for as typical palilalia. Though the vocalisations of this patient may be related to palilalia they remain difficult to classify given the limited categories of involuntary speech phenomena as described above.

The topography of the lesion as revealed by MRI may contribute to elucidate clinico-anatomical correlations of the involuntary vocalisations. The main lesion is in the pulvinar and the anterior-lateral to posterolateral thalamic nuclei with possible involvement of the pretectal area. Even though MRI is very sensitive to detect ischemic and haemorrhagic brain lesions, inter-

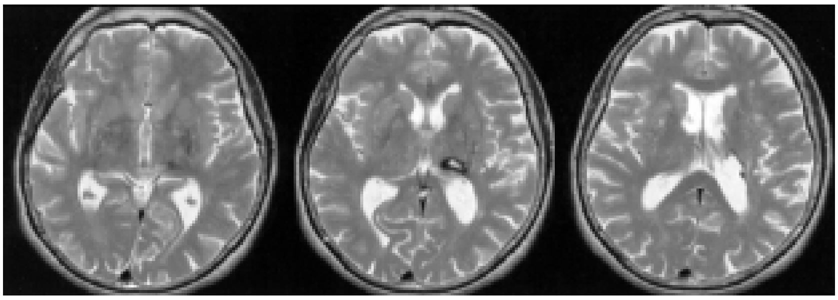

Fig. 1. MRI shows a left-sided haematoma predominantly located in the pulvinar extending to the lateroposterior thalamic nuclei and medio-caudally to the pretectal area (1.5 Tesla, dual fast spin echo method, TR/TZ 38/84 ms).

pretation of the specifity of functional neuroanatomical correlates is nevertheless limited.

Primarily based on the animal literature the role of the cingulate gyrus, the midbrain tegmentum and the periaqueductal grey matter have been suggested as anatomical sites involved in involuntary vocalisations [8,13]. Electric stimulation of the supplementory motor area produces complex vocalisations and jerks in humans [18]. However, in early reports on electric stimulation of the thalamus during stereotactic brain surgery the evocation of articulated phrases and words has been reported. For example, Schaltenbrand [20] described involuntary speech phenomena as a consequence of electric (mainly venterolateral) thalamic stimulation. This 'evoked compulsory speech' comprised complex sentences, monosyllabic yells and exclamations. Since contemporary reports on this compulsive speech phenomena are lacking the interpretation of these data requires some caution.

Lesions in the pulvinar and the posterolateral thalamus have been reported to cause transient dysphasic disorders as it was the case in our patient. Since these nuclei have extensive connections with language-relevant cortical structures a role in the recruitment and orchestration of the cortical areas subserving language functions has been proposed [16]. If this connectivity of the pulvinar and the posterolateral nuclei is related to the emergence of involuntary vocalisations in our patient can not be inferred from our clinical observations. Palilalia has been described following thalamic infarctions [11] and thalamic surgery in Parkinson's disease [22].

### 3.2. Movement disorder

Hemidystonia is a well recognized consequence of thalamic lesions. Particularly lesions of the posterior and the posterolateral thalamus as were present in the patient described above have consistently been associated with contralateral dystonia. As observed in our patient the dystonia has usually a delayed onset of several month [14].

In addition to right-sided hemidystonia the patient developed clonic arm jerks, mainly in the right arm, but also in the left arm. Bilateral clonic arm jerks have been described resulting from thalamic lesions (mainly bilateral paramedian lesions extending to midbrain structures) [6,14]. Lesions in the posterior nuclei of the thalamus may cause complex polymorphous jerky hyperkinetic syndromes variably described as tremor, myoclonus and chorea superimposed on hemidystonia [15]. The arm jerks of the patient described above may be part of the incompletely delineated spectrum of delayed hyperkinesias following lesions of the posterior thalamus [14,15].

Involuntary vocalisations and clonic jerks may stem from epileptic activity: For example, Bell [4] described a patient with rapid reiteration of meaningless syllables interpreted as phonemic jargon as part of neologistic speech automatism resulting from complex partial seizures. However, there is no evidence for seizure activity from EEG recording or clinical observations in the patient described above.

We suggest that in the case described above the co-occurence of hyperkinetic movement disorder and involuntary speech phenomena represents a release phenomenon resulting from the proximity of systems involved in motor and language functions in the posterior thalamus [17].

## References

[1] K. Abe, R. Yokoyama and S. Yorifuji, Repetitive speech disorder resulting from infarcts in the paramedian thalami and midbrain, *Journal of Neurology, Neurosurgery and Psychiatry* **56** (1993), 1024–1026.

[2] G.D.L. Bates, I. Lampert, M. Prendergast and A.E. van Woerkom, Klazomania: the screaming tic, *Neurocase* **2** (1996), 31–34.

[3] L. Benedeck, Zwangsmäßiges Schreien in Anfällen als postencephalitische Hyperkinese, *Zeitschrift für die gesamte Neurologie und Psychiatrie* (1925), 2–26.

[4] W.L. Bell, J. Horner, P. Logue and R.A. Radtke, Neologistic speech automatism during complex partial seizures, *Neurology* **40** (1990), 49–52.

[5] F. Boller, M. Albert and F. Dened, Palilalia, *British Journal of Disorders of Communication* **10** (1975), 92–97.

[6] P. Castaigne, F. Lhermitte, A. Buge, R. Escourolle, J.J. Hauw and O. Lyon-Caen, Paramedian thalamic and midbrain infarcts: clinical and neuropathological study, *Annals of Neurology* **10** (1981), 127–148.

[7] M. Critchley, Iterations of written and spoken speech: verbal tics, in: *Aphasiology and other aspects of language*, M. Critchley, ed, London: Edward Arnold Publishers, 1970, pp. 200–209.

[8] O. Devinsky, Neuroanatomy of Gilles de la Tourette Syndrome: Possible midbrain involvement, *Archives of Neurology* **40** (1983), 508–514.

[9] V.S.C. Fung, J.G.L. Morris, J. Leceister, Y.S. Soo, L. Davies, Clonic perseveration following thalamofrontal disconnection: a distinctive movement disorder, *Movement Disorders* **12** (1997), 378–385.

[10] J. Ghika, J. Bogousslavsky, J. Henderson, P. Maeder and F. Regli, The jerky dystonic unsteady hand: a delayed motor syndrome in posterior thalamic infarctions, *Journal of. Neurology* **241** (1994), 537–542.

[11] M. Ikeda and H. Tanabe, Two forms of palilalia: a clinicoanatomical study, *Behavioural Neurology* **5** (1992), 241–246.

[12] J. Jankovic, Phenomenology and classification of tics, *Neurological Clinics of North America* **15** (1997), 267–275.

[13] U. Jürgens and D. Ploog, On the neural control of mammalian vocalisation, *Trends in Neuroscience* **4** (1981), 135–137.

[14] M.S. Lee and C.D. Marsden, Movement disorders following lesions of the thalamus or subthalamic region, *Movement Disorders* **9** (1994), 493–507.

[15] G. Lera, O. Scipioni, S. Garcia, A. Cammarotta, G. Fischbein and O. Gershanik, A. combined pattern of movement disorders resulting from posterolateral thalamic lesions of a vascular nature: a syndrome with clinico-radiologic correlation, *Movement Disorders* **15** (2000), 120–126.

[16] S.E. Nadeau and B. Crosson, Subcortical aphasia, *Brain and Language* **58** (1997), 355–402.

[17] G.A. Ojemann, Common cortical and thalamic mechanism for language and motor function, *American Journal of Physiology* **246** (1984), R901–903.

[18] W. Penfield and L. Roberts, *Speech and brain mechanism*, Princetown University Press, Princetown, 1959.

[19] A. Pick, Die Palilalie, Ein Teilstück striärer Motilitätsstörungen, *Abhandlungen Neurologie Psychiatrie Psychologie* **13** (1921), 178–224.

[20] G. Schaltenbrand, The effects of stereotactic electrical stimulation in the depth of the brain, *Brain* **88** (1965), 835–840.

[21] A. Stracciari, M. Guarino, F. Cirignotta and P. Pazzaglia, Development of palilalia after stereotaxic thalamotomy in Parkinson's disease, *European Neurology* **33** (1993), 275–276.

[22] E. Tolosa and J. Peña, Involuntary vocalisations in movement disorders, in: *Advances in Neurology: Facial Dyskinesias*, J. Jancovic and E. Tolosa, eds, New York: Raven Press, 1988, pp. 343–363.

[23] Y. Yasuda, I. Akiguchi, M. Ino, H. Nabatabe and M. Kameyama, Paramedian thalamic and midbrain infarcts associated with palilalia, *Journal of Neurology, Neurosurgery and Psychiatry* **53** (1990), 797–799.
